# Communicating the Implementation of Open Notes to Health Care Professionals: Mixed Methods Study

**DOI:** 10.2196/22391

**Published:** 2021-08-16

**Authors:** Karin Jonnergård, Lena Petersson, Gudbjörg Erlingsdóttir

**Affiliations:** 1 Department of Business Administration Lund University Lund Sweden; 2 Department of Design Sciences Lund University Lund Sweden

**Keywords:** implementation, health care, electronic health records, communication strategy, eHealth, telemedicine, PAEHRs, Open Notes, professions, EHR

## Abstract

**Background:**

The literature on how to communicate reform in organizations has mainly focused on levels of hierarchy and has largely ignored the variety of professions that may be found within an organization. In this study, we focus on the relationship between media type and professional responses.

**Objective:**

The objective of this study was to investigate whether and how belonging to a profession influences the choice of communication media and the perception of information when a technical innovation is implemented in a health care setting.

**Methods:**

This study followed a mixed methods design based on observations and participant studies, as well as a survey of professionals in psychiatric health care in Sweden. The χ2 test was used to detect differences in perceptions between professional groups.

**Results:**

The use of available communication media differed among professions. These differences seem to be related to the status attached to each profession. The sense-making of the information appears to be similar among the professions, but is based on their traditional professional norms rather than on reflection on the reform at hand.

**Conclusions:**

When communicating about the implementation of a new technology, the choice of media and the message need to be attuned to the employees in both hierarchical and professional terms. This also applies to situations where professional employees are only indirectly affected by the implementation. A differentiated communication strategy is preferred over a downward cascade of information.

## Introduction

### Background

Since 2017, all residents of Sweden have been able to access their nonpsychiatric electronic health records (EHRs) through the internet, and thus have been able to read clinical notes. This “Open Notes” policy was first introduced in November 2012 by Region Uppsala, followed by Region Skåne in March 2014. In both regions, psychiatry was exempt because patient digital access was considered to be too sensitive. However, in 2015, Region Skåne included adult psychiatry in the service. This development is in line with the reasoning of the OpenNotes Project in the United States, which states that patients in psychiatric care should not be treated differently than other groups of patients in terms of their online access to EHRs [1-3].

Implementing new technical systems such as Open Notes in the public sector is often depicted as a complex process [4,5]. This is partly because activities in the public sector are affected by political and operational considerations, and are performed by both managers and street-level bureaucrats. Consequently, both an administrative and a professional hierarchy [6,7] organize activities. This complexity needs to be considered [8-11] for successful implementation of new technology, particularly with regard to how the implementation is communicated to employees [12,13]. When a reform directly affects professionals, this is obvious; however, when Open Notes was implemented in adult psychiatry in Region Skåne [14,15], the aim was to empower patients and the new policy was not expected to influence the work of health care professionals [16]. Because the professionals are required to input notes into the system and thus cannot opt out from the service, the implementation of Open Notes was regarded as a service related to their work tool, the EHR, which would enhance the transparency of their work [17], but was not supposed to influence the way they used the work tool. Health care professionals would only indirectly be affected by the implementation, which makes it interesting to reflect on how the implementation was communicated to them.

General reviews by King et al [18] and Cresswell and Sheikh [19] discuss technical, social, and organizational obstacles in the implementation of electronic health (eHealth) technology. In addition to issues related to the technology per se, they identify the great importance of acceptance of the technology by different professional groups active in health care [8,20-23]. Careful choices need to be made about how the implementation of different devices is communicated to professional groups [24]. In principle, this also applies to the communication of information about reforms that will affect the professionals only indirectly.

Media selection theory [25] has identified factors that are important for successful communication when reforms are implemented. These include features of the sender and the organization, the characteristics of the communication media and the messages, the receiver, and consideration of the receiver’s expected reaction. From a “perception management perspective,” the aim is to manage the receivers’ perceptions or sense-making of the information [24,26]. All information is subject to interpretation and its reception depends on the senders’ and receivers’ framing of the information [24,27], as well as the importance assigned to the matter [26,28]. However, it is also assumed that the receivers not only receive the information but also absorb it.

The perspectives of media selection theory and perception management are complementary. Media selection theories focus on the ability of an information channel to contribute rich information. It is commonly argued that channels that provide richer information should be available to managers at higher levels of the organization, especially in regard to equivocal tasks [29]. In addition, the need for coordination through communication is seen to increase along with the ambiguity of the implemented reform [30]. Research from a perception management perspective focuses on investigating the mode of implementation (hard/mixed/soft) [13] and the different stages in the diffusion of perceptions during implementation [26]. The perception management literature differs from media selection theory with respect to its emphasis that successful management of perception is context-dependent and may change over time. However, both perspectives assume the existence of an organizational hierarchy and task-oriented groups.

Professional groups are typically task-oriented. There is often a status hierarchy between different professions based on their different claims of jurisdiction [31] and on whether they are perceived as “full” professions or semiprofessions [32]. This status hierarchy is not always reflected in the formal organizational hierarchy. Thus, the theoretical assumptions described above may not always apply when implementing reforms in other contexts such as for health care organizations where the employees are professionals. Most of the research in both media selection theory and perception management perspective has dealt with formal hierarchical relationships within organizations without regard to the status of the professionals involved. Two related issues thus arise in relation to implementing an eHealth reform: (1) What media are best used to inform and change the perceptions of professional groups during the implementation phase? and (2) What aspects do the professionals perceive as important for the implementation? Far too little attention has been paid to these two issues. They may be mentioned, but have scarcely been investigated from a perception management perspective. Moreover, most research has focused on the implementation of reforms that are assumed to directly affect professionals. To our knowledge, no research has focused on a situation where professionals are indirectly affected by reforms.

The overall aim of this study was to investigate these two issues in relation to the implementation of Open Notes in adult psychiatry. Three research questions were formulated based on the results of a survey of professionals’ perceptions of the implementation process:

RQ1: Which strategies were used in the information and communication activities directed to professionals before the implementation of Open Notes?

RQ2: Do different professional groups demonstrate different patterns regarding the communication media through which they absorbed information?

RQ3: Which aspects did different professional groups perceive and prioritize as important in the communication?

This study builds on data from a previous mixed method study [33], focusing on (1) the strategies underlying the information and communication activities connected to the implementation of Open Notes in adult psychiatry in Region Skåne, (2) the channels through which the professionals received and absorbed information about the implementation, and (3) how essential they considered the information to be.

### Communication in the Processes of Implementation

A common approach to specify how information about technological innovations is diffused is to differentiate between dissemination and implementation. Dissemination refers to “active and planned efforts to persuade target groups to adopt an innovation” [34], whereas implementation refers to presenting information about the technical device’s functions and the way it will be integrated in the organization. According to Fidler and Johnson [35], implementation implies hierarchical power to implement the innovation, whereas dissemination does not. Considering Open Notes, the service is optional for the patients. The diffusion of the information to the patients can be characterized as dissemination. By contrast, the introduction of Open Notes to health care personnel can be viewed as a type of “indirect implementation,” as the reform did not aim to affect the work of the professionals, although it would increase the transparency between the patients and personnel. Open Notes was implemented in a typically top-down manner [36]: the decision was taken at a policy level and the implementation was seen as more of an administrative than a political process.

This type of implementation requires clear goals and change agents sympathetic to the goals. It presupposes that an organization has clear hierarchical relations and unifying organizational cultures. However, these presumptions are seldom valid in health care organizations, which are characterized as arenas for politicians, administrators, and professionals [6]. In such contexts, different groups may have different goals, and the hierarchical order may be blurred.

Markus and Pfeffer [37] mention three conditions that may obstruct implementation in organizations: (1) if the power distribution implicit in the reform does not match the existing power distribution in the organization, (2) if the language and symbols of the reform do not correspond to the dominant organizational paradigm and culture, and (3) if the goals and technology do not align with widely held goals and technology. Here, it should be noted that the hierarchy in professional groups is often assumed to be based on knowledge, with professionals being governed by professional norms and culture, and salient technology is regarded as directly connected to the work of the profession [38]. Both organizational and professional features could thus obstruct an indirect implementation such as Open Notes [9].

Rogers [10] argues that the complexity of a technological innovation is an important factor in the acceptance of reforms. If any part of the technology does not agree with the values of the adopters, or if the benefits are low or difficult to observe, the reform is likely to meet with resistance. In a case study on the implementation of diagnosis-related groups in Finnish health care, Lehtonen [11] drew conclusions in line with Rogers’ [10] assumptions. However, Lehtonen [11] also noted that early communication with clinical personnel and their involvement eased the implementation, as did freedom of choice regarding the degree to which the reform would be applied.

Communication between change management representatives and employees is crucial in any planned change process [12]. Mikkelsen et al [13] argue that the style of communication influences the motivation of the employees. Hard regulation from upper levels of the hierarchy may crowd out intrinsic motivation, while softer regulation may encourage employees’ own motivation and give them more positive perceptions of change. In addition, the views of what information should be shared and how it should be communicated may differ between management and employees. The greater the distance between the two groups, the less direct and rich the information the subordinate group receives will be. Employees have to rely on different levels of management to convey information to them. However, employee engagement and cooperation are key to success in the implementation process [39,40]. This is particularly true for organizations where employees are professionals and the implementation involves new technology [23,41].

A top-down implementation strategy implies programmatic change communication [42] (ie, a “telling and selling approach”). Russ [42] compares this approach to a “downward cascade of information about the change.” The advantage of programmatic change communication is the ability to disseminate quality information from the top of the organization to everyone, which gives the impression of equal and fair information. Programmatic change communication can lower the uncertainty surrounding a reform as well as the resistance to the innovation; it is also known to have the appeal of “high communication efficiency” [42].

Studies have shown that programmatic change communication is associated with problems such as alienation of employees, information overload, and growing cynicism regarding both the reform and top management [12,42,43]. Given its similarities to the hard regulation of innovation [13], programmatic change communication may be expected to influence the personnel’s intrinsic motivation, even though some research shows that this may not hamper the implementation as such [24]. However, the effects of programmatic change seem to be partly dependent on the channel of communication used. Common communication channels include general information meetings, posted information, and emailed information. In an early review of the field, Lewis [44] found that general information meetings and small informal discussions were the most commonly used channels for disseminating information about organizational changes. These results were confirmed by subsequent research [12,42]. Similarly, Ohemeng et al [26] found that workshops, seminars, training, one-on-one communication, and unit meetings were the main channels in attempts to manage perception.

As Open Notes was an indirect implementation, it is likely that programmatic change communication influenced both the way the employees perceived the information and the value they assigned to different information channels. Given the emphasis in earlier studies on “equal and fair” information, an important issue in the health care context is whether different professional groups perceived the communication to be in line with their professional norms and values.

## Methods

### Design

This study used a mixed methods approach with a sequential design [33]. There is thus a chronological link between the qualitative and quantitative data included in the study. First, we attended meetings, studied documents, and made observations to investigate the strategies behind the decisions about what information and communication professionals were deemed to need before implementation, and from which media they could access it. Thereafter, we designed a baseline survey and sent it to the professionals to investigate the actual use of media by different professional groups and how they perceived the reform.

### Empirical Material

#### Participation in Meetings

A multiprofessional working group was established in the autumn of 2013 in the division of psychiatry in Region Skåne. The group consisted of one professional from each of the four geographical administrative areas for adult psychiatry, one professional from child and youth psychiatry, one professional from forensic psychiatry, representatives of the communication department, technical developers, and representatives of patient organizations. The head physician led the working group and reported to management at the division of psychiatry. The working group held regular meetings to discuss and make decisions on the introduction, information, implementation, and development of Open Notes in the division of psychiatry. One of the authors of this paper attended and took field notes during 20 meetings from spring 2015 onward. These notes include summaries of important discussions and reflections on topics discussed at each meeting. The notes were used to define the strategies used as well as the perceptions of the reform.

#### Document Study

In spring 2015, representatives of the working group carried out a risk analysis to identify risks before implementing Open Notes in adult psychiatry. The risk analysis was a source of information when creating the questionnaire for the baseline survey and the strategies for information and communication.

#### Observation of Education Events

One of the authors attended eight educational events that were held for professionals in adult psychiatry before the implementation. Our aim was to gain knowledge about the content of the education and about the questions raised by professionals in the division of psychiatry in Region Skåne. Field notes, focusing on important questions and discussions, were used to document the eight observations.

### Baseline Survey

The baseline survey used in this study is based on the survey developed and implemented by the OpenNotes Project in the United States [45]. The original English version of the survey was translated and adapted to fit the Swedish context. The survey includes items concerning Open Notes and the work environment of the professionals. It was tested on two representative members of the working group and was then sent to all individuals employed in adult psychiatry in the region (N=3017). Four reminders were sent. As the survey closed 3 days before patients gained online access to their EHRs, all of the material in the baseline study was collected before the implementation.

The survey data reported in this article include demographic data on the participants’ professions and the results from two of the fixed-choice questions: one about the communication process and one about the implementation process. The results from one open-ended question about the information campaign are also included. These three questions were developed for the Swedish version of the survey. In the first question, health professionals were asked to report where they had received information about the reform. In conjunction with this fixed-choice question, there was an open-ended question in which the respondents could elaborate on how they perceived the information campaign. In the third question, respondents were asked to choose 5 out of 11 aspects they perceived as important for the implementation. They were then asked to rank these 5 aspects on a scale of 1 to 5, with 1 being the least important and 5 the most important. The responses to the open-ended question were subsequently categorized depending on whether the responses dealt with the content of the information provided or the form of the information.

### Ethics

The authors followed the guidelines on research ethics issued by the Swedish Research Council [46]. This study did not deal with any sensitive information, and according to Swedish regulations did not require ethical approval. Potential survey respondents were provided with information about the survey and its purpose in a prenotification email and a cover letter. The information stated that participation was voluntary and that withdrawal at any time without explanation was permitted, and further explained the confidentiality of the treatment and presentation of data.

### Data Analysis

The empirical material from the document studies, working group meetings, and educational events were analyzed and are presented as a narrative description in the Results section. The survey material was coded in Excel and imported into SPSS Statistics 23. The χ^2^ test was used to test differences between each profession and the rest of the professionals. All reported *P* values are two-sided. *P*<.01 was considered statistically significant.

## Results

### Demographics of the Survey Respondents

The response rate to the baseline survey was 28.87% (871/3017). The questionnaire was distributed to professionals in both permanent and temporary positions, which may have influenced the response rate negatively. Table 1 presents the demographics of the respondents and the entire population.

As the survey is a population study, it was important to investigate whether the 853 respondents were representative of the full population. The distribution of the different professions corresponds well with the overall percentage of professionals in each profession in the region. The survey population was compared with demographic information on all professionals in the field of adult psychiatry in Region Skåne. The comparison showed that the response rate was consistent for medical secretaries, a few percentage points lower for nurses and assistant nurses, and slightly higher for the other professional groups. All deviations were less than 10% (Table 1).

**Table 1:** Demographics of the respondents.

Characteristic		Survey respondents (N=871), n (%)	Population of the region (%)
**Professional affiliation^a^**			
	Doctor	133 (15.6)	11
	Medical secretary	76 (8.9)	8
	Psychologist	91 (10.7)	16^b^
	Nurse	228 (26.7)	28
	Assistant nurse	182 (21.3)	29
	Sociotherapist^c^	90 (10.6)	—^d^
	Other	53 (6.2)	N/A^e^
**Gender^f^**			
	Male	223 (26.2)	—^g^
	Female	628 (73.8)	—^g^

^a^853 of the 871 respondents answered the question about their professional affiliation.

^b^Social workers, occupational therapists, physical therapists, and psychologists are included in the same group for the total region.

^c^Includes social workers, occupational therapists, and physiotherapists.

^d^Included in the psychologist category.

^e^N/A: not applicable.

^f^851 of the 871 respondents answered the question about their gender.

^g^No information available.

### Communication Channels

This section deals with the first research question, which concerns the strategies underlying the information and communication activities prior to implementation. As mentioned in the Methods section, a multiprofessional working group was established in 2013. The group comprised professionals from different parts of the region and was intended to be representative of the professions as well as the different geographic areas.

The working group had regular meetings to discuss, plan, and make decisions on the introduction and implementation of Open Notes in adult psychiatry. Educational events were also planned. As this was the first psychiatric setting in Sweden to implement Open Notes, many questions had to be addressed before the service could be implemented. The working group decided that a risk analysis was needed.

A new group consisting of employees from different professions and a few members of the working group was asked to carry out the analysis. The risk analysis was performed at the beginning of 2015. The aim was to identify risks to patient safety in connection with the implementation and use of Open Notes in psychiatry, and to identify possible risks for patients’ relatives and professionals. Another aim was to identify the benefits of Open Notes for patients and health care. The risk analysis group report mentioned the need for information to be given to professionals to safeguard patients. The analysis suggested that this information should be available on the intranet, where a site was developed and continuously updated. However, the working group realized that this was not sufficient since they became aware that some professionals felt that they had not received any information about the implementation. Consequently, the working group decided that more communication channels were needed. It was considered crucial to use all suitable communication channels to make professionals aware of the change and ensure that they understood the planned implementation. The choice of media was based on previous experience (ie, “how we used to do it”) rather than on the specific characteristics of Open Notes.

Before the implementation, the following communication and information activities were carried out: (i) information was posted on Region Skåne’s intranet about the implementation, (ii) information emails were sent to professionals by managers, (iii) information/education in two identical 1.5-hour sessions (one morning and one afternoon session) was provided in each of the four geographic areas in the spring of 2015, and (iv) information/communication was provided at workplace meetings.

Information/communication was also available at professional staff meetings arranged by unions. The first two activities were based on a push strategy and a one-way transmission model of communication, whereas the last two and the union meetings enabled opportunities for sense-making through dialog and feedback [26].

The working group considered the information/education events the most important change communication effort because they enabled more symmetrical communication. These events were used to inform health professionals about the implementation and give them opportunities to raise questions, participate, and become involved in their workplace. In other words, the working group aimed to change the professionals’ perceptions by applying both rich information strategies [47], which provided a base for interactions and collective interpretations [22], and less rich information strategies.

In total, approximately 300 professionals attended the eight information/educational events in late April and early May 2015. The project manager for the implementation of Open Notes in Region Skåne was responsible for each event, together with a local representative from the working group. Thus, different individuals were responsible for the information at different geographic locations, which resulted in slightly different presentations of Open Notes at each event. The events consisted of a video with general information about Open Notes, followed by a PowerPoint presentation about the decision-making process prior to implementation, the advantages of Open Notes, and the identified risks. Both the video and the PowerPoint introduced the reform rather superficially. There was also a demonstration of what the interface would look like for patients. There were opportunities to ask questions and discuss the implementation.

The professionals’ questions were mainly about the technical prerequisites, the new routines with confidentiality checks when an entry was written in the health record, how information from relatives should be handled safely, and the need for more information about the implementation. As neither the full technical prerequisites nor the implementation date were clear when the events took place, it was not possible to answer some of the questions that the professionals considered important. This presented a major communication challenge.

In summary, patient security was the main focus of the working group, and the information given to professionals was in accordance with this focus. The media used were routinely chosen and the opportunity for “richer” information was limited by the state of the development of the technology at the time of the information/educational events.

### Use of Different Communication Channels Among Professionals

This section deals with the second research question, which concerns the media used to inform and change the perceptions of professional groups prior to the implementation. In particular, we focus on how well management was able to communicate information to professionals in adult psychiatry using these media.

As different media were available, the focus was on the media the professions normally used. Table 2 presents the results, showing that the respondents received and absorbed information through a variety of channels. It is important to note that the questionnaire allowed the respondents to choose multiple responses; therefore, the percentages in the total number of responses column in Table 2 add up to more than 100%. There were 1750 responses to this question. Responses from those who did not state their profession were excluded. The results show that media that allowed for dialog and rich information predominated. Overall, 49% of the respondents stated that they received information at a workplace meeting, 25% from informal conversations with colleagues, and 14% at an education event held in the spring of 2015. The unidirectional channel of the intranet was the medium of information for 40% of the professionals, and 16% received information through mass media. The classification of email under dialog media depends on whether the receiver perceived it possible to respond by asking questions; 38% indicated that they received information through email. It is noteworthy that 7% of the professionals claimed that they had not received any information.

Considering the differences among individual professions, doctors stood out as obtaining information through professional meetings and informal conversations substantially more than the rest of the respondents, and significantly less through workplace meetings. In other words, their communication largely took place through rich channels with the ability to shape perceptions. By contrast, the medical secretaries informed themselves through the intranet significantly more than the total number of respondents (ie, they used unidirectional, less rich channels). Psychologists and sociotherapists received information through workplace meetings to a significantly higher degree. In addition, psychologists gained information through informal conversations, whereas sociotherapists gained information through an education event. Assistant nurses took part in education events to a significantly lower degree and used email significantly more often when compared to the total number of respondents. The nurses, assistant nurses, and sociotherapists gained significantly less information through meetings with fellow professionals than the total number of respondents.

In conjunction with the fixed-choice question, there was an open-ended question where the respondents could elaborate on how they perceived the information campaign. Among the 871 professionals, 92 (10.6%) responded with free-form text to the question, “Do you have any further comments on the information surrounding Open Notes?” The free-form text answers dealt either with the content of the information or the way the information was transferred. First, there were requests for a different type of educational event with more detailed information about such matters as the technical prerequisites for the Open Notes system and legal issues surrounding the new transparency of the contents of health records. There were also requests for clearer and more substantial content beyond information about the fact that Open Notes was going to be implemented in adult psychiatry. Second, there were comments about the information process, with a desire for more dialog and two-way communication for the professionals before and during the implementation process. Some also wished that the educational events had been more frequent and held in more locations.

**Table 2:** Responses to the statement “I have received information about online patient access to their electronic health records in adult psychiatry through...(you can choose several responses to this statement)” (N=1750).^a,b^

Information source	Doctor (N=132), n (%)	Medical secretary (N=73), n (%)	Psychologist (N=91), n (%)	Nurse (N=224), n (%)	Assistant nurse (N=180), n (%)	Sociotherapist (N=89), n (%)	Total responses, n (%)
Workplace meeting	38 (28.8)^c^	36 (49.3)	60 (65.9)^c^	120 (53.6)	80 (44.4)	57 (64.0)^c^	414 (49)
Intranet	45 (34.1)	38 (52.1)^c^	35 (38.5)	89 (39.7)	63 (35.0)	42 (47.2)	342 (40)
Email	59 (44.7)	29 (39.7)	27 (29.7)	77 (34.4)	84 (46.7)^c^	26 (29.2)	320 (38)
Informal conversation	47 (35.6)^c^	19 (26.0)	32 (35.2)^c^	54 (24.1)	32 (17.8)	18 (20.2)	211 (25)
Mass media	29 (22.0)	6 (8.2)	13 (14.3)	44 (19.6)	28 (15.6)	8 (8.9)	134 (16)
Education event	17 (12.9)	16 (21.9)	12 (13.2)	25 (11.2)	15 (8.3)^c^	22 (24.7)^c^	122 (14)
Professional meeting	66 (50.0)^c^	5 (6.8)	7 (7.7)	10 (4.5)^c^	3 (1.7)^c^	1 (1.1)^c^	110 (13)
Social media	4 (3.0)	4 (5.5)	1 (1.1)	11 (4.9)	9 (5.0)	2 (2.2)	35 (4)
No information	8 (6.1)	5 (6.8)	7 (7.7)	18 (8.0)	19 (10.6)	2 (2.2)	62 (7)

^a^Note to interpret the percentages in this table: As an example, 28.8% of doctors stated that they received information from workplace meetings, 34.1% of doctors replied intranet, 44.7% of doctors replied email, and so on.

^b^Since multiple responses were possible, the percentages are above 100%.

^c^*P*<.01 compared with all other professional groups.

### Importance of Different Aspects of the Implementation Process

The third research question was related to the aspects the professionals perceived as important for the implementation. Ohemeng et al [26] describe this as “the third step…where the stakeholders attempt to make sense by trying to figure out the meaning of the proposed vision, and revising their understanding.” Eleven aspects of the implementation were listed, and the professionals were asked to rank the five most important aspects on a scale of 1 to 5 (with 1 as the least important and 5 as the most important). Table 3 summarizes the total number of respondents who mentioned an aspect, the total ranking scores, and the mean. Table 4 shows the results for the different professions in terms of the percentage of professionals who mentioned the aspect, the mean of the ranking scores given by the professionals who mentioned the aspect, and the importance rank the professionals assigned to the aspect.

Overall, the most frequently chosen aspect was “Evaluation of the Open Notes service,” although “Patient safety” had the highest total score and also the highest mean value (see Table 3). It is interesting to note that the aspect receiving the lowest score was “A support line for professionals.” This aspect also had the lowest total score and the lowest mean value. However, the differences between the professional groups were small (see Table 4). Reviewing the aspects ranked as the five highest (according to their means) shows that all professional groups included “Patient safety,” “Information to professionals,” and “Professionals’ participation in the process.” The medical secretaries diverged the most from the general picture in that they ranked “Information to professionals” as the most important, whereas “Information to patients” was outside of the five highest means for this group. Again, note that the differences were small and nonsignificant. Both the medical secretaries and the doctors included “System reliability” among the first five aspects. However, the largest difference is that the importance of “Patient safety” only ranked in the third priority for the medical secretaries, whereas it was ranked of primary importance for all other groups. A tentative conclusion would be that perceptions differ between health care personnel and administrative personnel.

**Table 3:** Total scores of importance of different aspects of the implementation.

Aspects	Answers (n)	Total score	Mean
Information to management	201	532	2.65
Information to professionals	399	1334	3.34
Education for professionals	421	1383	3.29
Professionals’ participation in the process	329	1025	3.12
A support line for professionals	156	388	2.49
Information to patients	477	1526	3.20
Patient safety	503	1814	3.61
A support line for patients	246	663	2.70
System reliability	283	823	2.91
System fitness for use and clarity	316	801	2.53
Evaluation of the Open Notes service	536	1445	2.70

**Table 4:** Responses to the statement “Choose five aspects you perceive as important for the Open Notes implementation. Rank the most important 5, the next most important 4, etc, down to 1.”

Aspects			Doctors		Medical secretaries		Psychologists		Nurses		Assistant nurses		Sociotherapists, etc
**Information to management**													
	%	19		29		23		20		30		20	
	Mean	2.36		2.86		2.52		2.76		2.82		2.11	
	Importance	—^a^		—		—		—		—		—	
**Information to professionals**													
	%	33		49		53		50		47		50	
	Mean	3.36		3.68		3.15		3.46		3.43		2.82	
	Importance	2		1		4		2		3		—	
**Education for professionals**													
	%	47		55		50		49		47		54	
	Mean	2.95		3.60		3.33		3.23		3.45		3.27	
	Importance	—		2		2		4		2		3	
**Professionals’ participation in the process**													
	%	39		29		45		41		37		38	
	Mean	3.19		2.91		3.29		3.01		3.01		3.21	
	Importance	3		4		3		5		5		4	
**A support line for professionals**													
	%	23		17		15		16		19		18	
	Mean	2.61		2.38		2.71		2.58		2.47		2.31	
	Importance	—		—		—		—		—		—	
**Information to patients**													
	%	47		58		20		61		51		58	
	Mean	3.06		2.73		3.14		3.27		3.27		3.38	
	Importance	5		—		5		3		4		2	
**Patient safety**													
	%	59		53		63		59		59		62	
	Mean	3.63		3.40		3.88		3.52		3.48		3.77	
	Importance	1		3		1		1		1		1	
**A support line for patients**													
	%	39		25		20		28		28		31	
	Mean	2.67		2.84		2.44		2.86		2.80		2.43	
	Importance	—		—		—		—		—		—	
**System reliability**													
	%	35		26		32		35		25		39	
	Mean	3.15		2.80		2.52		2.94		2.76		2.49	
	Importance	4		5		—		—		—		—	
**System fitness for use and clarity**													
	%	41		25		39		37		35		39	
	Mean	2.69		2.63		2.34		2.58		2.47		2.43	
	Importance	—		—		—		—		—		—	
**Evaluation of the Open Notes service**													
	%	74		46		70		60		55		66	
	Mean	2.91		2.74		2.66		2.48		2.62		2.92	
	Importance	—		—		—		—		—		5	

^a^Not ranked in the top 5.

## Discussion

### Principal Findings

The Open Notes reform was novel both as a technological innovation and because the receivers were defined as the patients rather than the organization or its professionals. The focus on patients and patient security therefore dominates the implementation [48]. The risk analysis performed by the working group amplified the importance of the patient. Informing the professionals was perceived as a step in the strategy to ensure patient safety, implying that other issues such as the professionals’ work situation were of secondary concern when formulating the communication strategy. The communication strategies selected by the working group consisted both of richer media such as education and meetings, and of less rich media such as email and intranet pages. As mentioned above, the implementation was a “telling and selling” process focused on giving information, even though the education and meeting may also be viewed as a way to change perceptions.

Given the focus on the patient as a receiver of the reform, the emphasis on giving information rather than changing perceptions is not surprising. The communication strategies were uniform; that is, all media sources were intended to inform all groups of professionals. Where perceptions might be changed (eg, at education events), the information was provided through a video and PowerPoint presentation with general content. However, the presentations varied depending on the person leading the discussion, who was responsible for presenting the information, as well as on the stage of technological development of the Open Notes service at that time. It appears that the choice of communication strategies was based on the perception that the reform was unambiguous [30] and would have low technical complexity [10] in the eyes of the professionals. Because the working group did not view the reform as opposed to the values of the adapter [10], there was no perceived need to distinguish between professional groups.

These features of the communication strategy are not surprising given that the effect of the reform on health professionals was viewed as indirect; they were viewed as intermediaries rather than receivers of the reform. This supports our finding that although the implementers did not ignore the professionals’ need for information and the need to change their perceptions, these concerns were regarded as background concerns rather than as key issues.

The pattern of reception of the information indicates that dialog media in workplace meetings were the most common modes of absorbing information, whereas intranet and email were the second most commonly used media. However, when considering the pattern across professions, a scale of media use connected to social status and workplace organization becomes visible. The scale is bookended by the doctors and the medical secretaries. The latter group relied mainly on nondialogic media, whereas the doctors primarily used dialogic information channels and mostly gained their information at professional meetings (ie, through their peers) or through informal conversations. The use of media by other professions was distributed between these two groups in a way that largely reflects the traditional order of professional status. However, work organization also appears to have an impact. For example, to obtain information at meetings, one has to participate, and for professional meetings to be important, a strong union is needed. Participation in workplace meetings is easier to achieve because the professionals must attend and because there is a sense of belonging to the workplace. However, doctors often obtained their information at internal professional meetings. As the union organized these meetings, management influence over the information given was low. This raises the issue of whose perception of the reform is diffused, and how this affects the implementation.

The use of nondialogic media was rather high for all personnel groups. This is not surprising as computers are a standard work tool in the health care sector in Sweden. The routine use of computers means that media distributed by computer are easily accessible, making obtaining new information part of the everyday routine of accessing information at the workplace. Previous research emphasizes that nondialogic media imply less rich information. It is likely that email is often one-way communication and is perceived as a hard regulation for diffusing information [13,24]. It follows that information transmitted by email shapes perceptions to a lesser degree and infuses a sense of incapacity.

Despite all of the different communication channels used in the communication campaign, the free-form text answers revealed that information had not reached all professionals or, at least, they had not all received the necessary information. It is also noteworthy that 7% of the survey respondents stated that they had received no information at all, despite all efforts made by management. According to the data (see Table 2), assistant nurses were the largest group in this category, with 11% responding that they had received no information. They were also the largest group receiving information by email, whereas primary sources of information for the other professional groups were either workplace or professional meetings. The reason for this finding is outside the scope of this study, but it indicates that either work organization or professional status is important when using information channels. In addition, the answers to the open-ended question indicate a need for more dialogic media and more substantive information. This indicates the importance of using rich media when innovations are perceived as complex by the personnel (even if not by the implementers).

The medical secretaries stood out in regard to the aspects perceived as important in the implementation process. In contrast to all other personnel groups who ranked “Patient safety” as the highest on average, the medical secretaries showed higher average rankings for both “Information to professionals” and “Education for professionals.” However, these differences were small. Overall, one can discern a tendency to emphasize aspects related to patients and personnel rather than technical concerns. This indicates that the social and organizational aspects of implementing Open Notes are the salient issues for the personnel in psychiatric care. The sense-making showed primary consideration for the patient, followed by the professionals. This emphasis on the patient is not surprising given that client care is the normative basis for most professions [31,38]. It is likely that the value of “patient safety” promoted by the working group was already embedded in the professionals’ norm system. The reaction from the respondents is thus not surprising, but this finding enriches research from a perception management perspective by introducing consideration of the likely effect of the professional norms of the receiver on their sense-making of information. These results also suggest that when formulating a message, attention should be paid to the values and social aspects that are important to the receiver rather than to technical information such as system features. Rich information in this context does not simply imply “a lot of information” but rather information that agrees with or affects with the interpretative frames connected to the professions.

From the perspective of perception management, the implementation of Open Notes can be viewed as a hard regulation. The health care personnel had no option but to accept the implementation in the form decided by management. When the professionals “made sense” of the implementation, it was consequently not the technical issues that were in the forefront. Instead, aspects related to patient safety and in-depth information for the professions were salient. This can be interpreted as perception management having succeeded in “selling” the solution, but awakened concerns connected to professional norms and values while doing so.

### Limitations

This study has several limitations. First, the response rate to the web questionnaire was only 28.86%. One explanation may be that this was a full population study and some employees were not working during the time when it was possible to answer the survey. Nevertheless, the group distribution among the respondents corresponds well with the percentage of employees in each profession, which indicates that we have good representation of all professional groups. As Open Notes was implemented later in adult psychiatry than in other areas of health care in the region, it is also possible that some of the respondents gained knowledge about the reform from other sources. The items in our survey do not cover this.

The majority of respondents reported that they had gained information from more than one medium. Since the respondents specified more than one medium in the survey, we do not know whether these media complemented or substituted for each other. The study would have been improved if we had also asked which media the responders found to be the most important. This would had given us a firmer base to discuss the importance of media type when interpreting reforms.

### Conclusions

This paper makes several contributions. The first is the empirical evidence that different groups of professionals absorb information through different channels when informing themselves about reforms. Our working hypothesis was that health care organizations have “double hierarchies,” and that these have to be considered when communicating implementations. The results of this study largely confirm this hypothesis. A main conclusion of the study is that professional association matters both for the choice of information media and for evaluating aspects of the message that comes from a higher level of an organization. Those in groups that are considered “full professions” with high professional status prefer to be informed among their peers, whereas semiprofessionals find other ways to become informed. This difference may be because full professions often have a more stable professional identity and more opportunities to meet fellow professionals. This observation adds to the importance in media selection theory of not only considering the hierarchical levels of the organization but also the different status of the professionals in the organization. We also discerned minor differences between professional groups regarding which issues they perceived to be important in implementing reforms. From the point of view of communications practitioners, this finding implies that communication strategies may be more successful when they combine common information with strategies directed toward different professionals for communicating indirect implementations.

A second contribution is the finding that professional status alone does not determine the choice of information channel. The pattern of media use described is many-sided. Most professional groups mention email or intranet as one source of information. These are channels that are routinely used for diffusing information in an organization. There are also indications that the work organization is important, such as participation in workplace meetings or educational events. Thus, a communication strategy has to consider professional diversity and workplace organization, as well as existing information paths to fully reach out to the receivers.

The third contribution is defining the implementation of Open Notes as an example of an indirect implementation. This feature influenced the perception of the management. From the point of view of the working group, health professionals, however significant, were perceived largely as “tools” to achieve the primary aim of the reform: patient empowerment without risking patient safety. This perception influenced both the channels of communication and the information content. The information to professionals was presented in a routine manner and was sometimes incomplete, possibly because management did not believe that the reform would affect health professionals. However, any implementation may involve new roles for various parties regardless of whether they are directly or indirectly affected. In this case, for example, the professionals will be meeting more “empowered” patients. An alternative approach is to pay attention to the effect of the implemented reforms on both those directly affected and those indirectly affected. It is likely that implementation of reforms that indirectly affect professions will be more common in the future, as the discourse of patient empowerment is taken up in other areas. However, as a rather new phenomenon, more research is needed both about how indirect implementation of reforms may affect professionals and about the kind of information that is important to ease the implementation.

In conclusion, we have compared the communication strategy regarding choice of media and the most important aspect of the reform as perceived by the receivers. Our main conclusion is that there is a link between the management’s (ie, work group’s) perception of the main receiver of the reform (here, the patients), and the communication strategy used in the health organization. By contrast, the reception of the information seems to depend on the mix of professions in the organization and their professional norms, as well as on the work organization and routine paths used to disseminate information. However, research on indirect implementation is still in its early stages. More research is needed before these relationships are fully understood. Finding good strategies for providing information to different professional groups will be valuable when communicating with those indirectly affected by an implementation.

How various aspects of communication interact is an important issue for future research. However, the complexity of the use of media revealed in this study indicates that, in general, a multimedia approach may more easily succeed than a single-medium approach.

## Abbreviations

eHealth: electronic health
EHR: electronic health record

## References

[1] Kahn MW, Bell SK, Walker J, Delbanco T (2014). A piece of my mind. Let's show patients their mental health records. JAMA.

[2] Walker J, Kahn MW, Delbanco T (2014). Transparency in the delivery of mental health care--reply. JAMA.

[3] Dobscha SK, Denneson LM, Jacobson LE, Williams HB, Cromer R, Woods S (2016). VA mental health clinician experiences and attitudes toward OpenNotes. Gen Hosp Psychiatry.

[4] Goldfinch S (2007). Pessimism, computer failure, and information systems development in the public sector. Public Admin Rev.

[5] Stewart J, O'Donnell M (2007). Implementing change in a public agency. Intl Jnl Public Sec Manag.

[6] Brunsson N, Sahlin-Andersson K (2016). Constructing organizations: the example of public sector reform. Organiz Stud.

[7] Lipsky M (1980). Street-level bureaucracy: dilemmas of the individual in public services.

[8] Cucciniello M, Lapsley I, Nasi G, Pagliari C (2015). Understanding key factors affecting electronic medical record implementation: a sociotechnical approach. BMC Health Serv Res.

[9] Moullin JC, Sabater-Hernández D, Fernandez-Llimos F, Benrimoj SI (2015). A systematic review of implementation frameworks of innovations in healthcare and resulting generic implementation framework. Health Res Policy Syst.

[10] Rogers E (2003). Diffusion of Innovations.

[11] Lehtonen T (2007). DRG-based prospective pricing and case-mix accounting—Exploring the mechanisms of successful implementation. Manag Account Res.

[12] Lewis LK (2006). Employee perspectives on implementation communication as predictors of perceptions of success and resistance. West J Commun.

[13] Mikkelsen MF, Jacobsen CB, Andersen LB (2015). Managing employee motivation: exploring the connections between managers’ enforcement actions, employee perceptions, and employee intrinsic motivation. Int Public Manag J.

[14] Petersson L, Erlingsdóttir G (2018). Open Notes in Swedish psychiatric care (part 1): survey among psychiatric care professionals. JMIR Ment Health.

[15] Petersson L, Erlingsdóttir G (2018). Open Notes in Swedish psychiatric care (part 2): survey among psychiatric care professionals. JMIR Ment Health.

[16] Erlingsdóttir G, Lindholm C (2015). When patient empowerment encounters professional autonomy: The conflict and negotiation process of inscribing an eHealth service. Scand J Public Admin.

[17] Erlingsdóttir G, Petersson L, Jonnergård K (2019). A theoretical twist on the transparency of Open Notes: qualitative analysis of health care professionals' free-text answers. J Med Internet Res.

[18] King G, O'Donnell C, Boddy D, Smith F, Heaney D, Mair FS (2012). Boundaries and e-health implementation in health and social care. BMC Med Inform Decis Mak.

[19] Cresswell K, Sheikh A (2013). Organizational issues in the implementation and adoption of health information technology innovations: an interpretative review. Int J Med Inform.

[20] Murray E, Burns J, May C, Finch T, O'Donnell C, Wallace P, Mair F (2011). Why is it difficult to implement e-health initiatives? A qualitative study. Implement Sci.

[21] Boonstra A, Broekhuis M (2010). Barriers to the acceptance of electronic medical records by physicians from systematic review to taxonomy and interventions. BMC Health Serv Res.

[22] Barrett AK, Stephens KK (2016). The pivotal role of change appropriation in the implementation of health care technology. Manag Commun Quart.

[23] Eriksson-Zetterquist U, Lindberg K, Styhre A (2009). When the good times are over: professionals encountering new technology. Hum Relat.

[24] Grøn CH (2018). Perceptions unfolded: managerial implementation in perception formation. Int J Public Sec Manag.

[25] Timmerman CE (2016). Media selection during the implementation of planned organizational change. Manag Commun Quart.

[26] Ohemeng FLK, Amoako Asiedu E, Obuobisa-Darko T (2018). Giving sense and changing perceptions in the implementation of the performance management system in public sector organisations in developing countries. Int J Public Sec Manag.

[27] Deline MB (2018). Framing resistance: identifying frames that guide resistance interpretations at work. Manag Commun Quart.

[28] Martine T, Cooren F, Bénel A, Zacklad M (2015). What does really matter in technology adoption and use? A CCO approach. Manag Commun Quart.

[29] Donabedian B, McKinnon SM, Bruns WJ (2016). Task characteristics, managerial socialization, and media selection. Manag Commun Quart.

[30] Donabedian B (2006). Optimization and its alternative in media choice: a model of reliance on social-influence processes. Inf Society.

[31] Abbott A (1988). The system of professions: an essay on the division of expert labor.

[32] Brante T (2013). The professional landscape: the historical development of professions in Sweden. Prof Prof.

[33] Teddlie C, Tashakkori A (2009). Foundations of mixed methods research: integrating quantitative and qualitative approaches in the social and behavioral sciences.

[34] Greenhalgh T, Robert G, Macfarlane F, Bate P, Kyriakidou O (2004). Diffusion of innovations in service organizations: systematic review and recommendations. Milbank Q.

[35] Fidler LA, Johnson JD (1984). Communication and Innovation Implementation. Acad Manag Rev.

[36] Matland R.E (1995). Synthesizing the implementation literature: The ambiguity-conflict model of policy implementation. J Public Admin Res Theory.

[37] Markus M, Pfeffer J (1983). Power and the design and implementation of accounting and control systems. Account Organiz Soc.

[38] Freidson E. (2001). Professionalism: the third logic.

[39] Lewis L (2011). Organizational change: creating change through strategic communication.

[40] Klein SM (1996). A management communication strategy for change. J Org Change Manag.

[41] Constantinides P, Barrett M (2006). Negotiating ICT development and use: The case of a telemedicine system in the healthcare region of Crete. Inf Organ.

[42] Russ TL (2008). Communicating change: a review and critical analysis of programmatic and participatory implementation approaches. J Change Manag.

[43] Qian Y, Daniels TD (2008). A communication model of employee cynicism toward organizational change. Corp Comm.

[44] Lewis LK (2016). Disseminating information and soliciting input during planned organizational change. Manag Commun Quart.

[45] Walker J, Leveille SG, Ngo L, Vodicka E, Darer JD, Dhanireddy S, Elmore JG, Feldman HJ, Lichtenfeld MJ, Oster N, Ralston JD, Ross SE, Delbanco T (2011). Inviting patients to read their doctors' notes: patients and doctors look ahead: patient and physician surveys. Ann Intern Med.

[46] Swedish Research Council Good Research Practice.

[47] Daft RL, Lengel RH, Trevino LK (1987). Message equivocality, media selection, and manager performance: implications for information systems. MIS Quart.

[48] Nøhr C, Parv L, Kink P, Cummings E, Almond H, Nørgaard JR, Turner P (2017). Nationwide citizen access to their health data: analysing and comparing experiences in Denmark, Estonia and Australia. BMC Health Serv Res.

